# The Perception of Family Function by Adolescents with Epilepsy in a Rural Nigerian Community

**DOI:** 10.1155/2014/959274

**Published:** 2014-11-25

**Authors:** Edwin E. Eseigbe, Folorunsho T. Nuhu, Taiwo L. Sheikh, Sam J. Adama, Patricia Eseigbe, Okechukwu J. Oguizu

**Affiliations:** ^1^Department of Paediatrics, Ahmadu Bello University Teaching Hospital, Zaria 810001, Nigeria; ^2^Federal Neuropsychiatric Hospital (FNPH), Kaduna, Nigeria; ^3^Department of Paediatrics, 44 Nigerian Army Reference Hospital, Kaduna, Nigeria; ^4^Department of Family Medicine, Ahmadu Bello University Teaching Hospital, Zaria 810001, Nigeria

## Abstract

The family plays a significant role in epilepsy management in sub-Saharan Africa and how this role is perceived by persons with epilepsy could influence epilepsy outcomes. The objective of the study was to assess perception of family function by adolescents with epilepsy (AWE). The sociodemographic and epilepsy characteristics of AWE in a rural Nigerian community were assessed and the Family APGAR tool was used in assessing their perception of satisfaction with family functioning. Adolescents (*n* = 1708) constituted 26% of the community's population and 18 (10.5/1000) had epilepsy. The AWE age range was 11–19 years (mean 16.7 ± 2.6 years) with a male preponderance (15, 83.3%). The family was the only source of care. Family dysfunction (Family APGAR Score <7) was indicated by 15 (83.3%) of the AWE. The strongest perception of family function was in adaptability while the weakest was with growth. The indication of family dysfunction was significant (*P<*0.05) in the older (age 14–19 years) AWE when compared with the younger AWE (11–13 years) in the study. Most of the AWE indicated living in a dysfunctional family setting. The study highlights the need to address the role of the family in the provision of comprehensive epilepsy care.

## 1. Introduction

Epilepsy affects 70 million persons worldwide and a high age specific incidence is associated with the adolescent population [1, 2]. The worldwide mean prevalence of epilepsy is 8.9/1000 [3]. Epilepsy prevalence rates of 4.3–26.1/1000 have been reported from several child and adolescent populations globally [4]. A significant proportion of those with the disease live in low and middle income countries (LMICs) where there is limited access to effective treatment [1, 2]. Poor outcomes of epilepsy such as stigma, depression, poor quality of life, and even death have been associated with epilepsy in the adolescent [5–11]. Ignorance, poor sociocultural epilepsy perspectives, weak health systems, epilepsy treatment gap, weak social support system, and family dysfunction are some factors that are contributory to these poor epilepsy outcomes [1–3, 5–12].

Studies have shown that adolescent perspectives on epilepsy have significant impact on the outcomes of the disease [7–10, 13, 14]. Most of these studies have been on awareness and knowledge of epilepsy and attitudes towards and the Health Related Quality of Life (HRQOL) in adolescents with epilepsy (AWE). Reports from these studies have indicated poor academic achievement, a comparatively lower HRQOL than that of the general population, lowered self-esteem, depression, poor attitudes, and a limited knowledge of epilepsy among AWE [7–10, 13, 14]. The identification of these perspectives facilitates the development and institution of interventions that could ensure adequate access to needed therapy.

In most of the LMICs statutory care or support for adolescents is lacking in health and social systems resulting in an almost total dependence on family settings for care [15]. In the African region weaknesses in health systems, stigma, and communal isolation associated with epilepsy entrust care of persons with epilepsy primarily to families and in family settings [2]. Consequently the effective functioning of the family in the region would have implications on the outcome of chronic diseases such as epilepsy.

Family functioning has been defined as the way in which family members communicate, relate, and maintain relationships among each other, as well as the way they make decisions to solve problems [16]. Satisfactory family functioning has been associated with positive outcomes in epilepsy [12, 17]. Also factors that disrupt family functioning such as low socioeconomic status, poor epilepsy awareness, stress, and parental psychopathologies have been associated with poorer epilepsy outcomes [1–3, 8, 17]. Thus outcomes in AWE in LMICs could be significantly influenced by the quality of family functioning.

Overall, the adolescent period provides opportunities and vulnerabilities with its wellbeing hinging on the development of help seeking behavior of the adolescent [15]. Perception of others and helping institutions, as helpful and trustworthy, has been identified as one of the factors that influence the help seeking behavior of adolescents [15]. Therefore in settings such as ours, where family support is the main source of care for adolescents with epilepsy, understanding adolescents' perception of this support and other family functions could have significant impact on epilepsy outcomes. Reports on AWE perception of family functioning from Nigeria are scarce. Adewuya et al. [7] reported perception of high family functioning among a majority of AWE in an urban Nigerian setting. To provide more insight on the perception of family functioning by AWE, and its impact on epilepsy outcomes, more studies are required.

The objective of the study was to assess perception of family function by AWE in a rural Nigerian community.

## 2. Materials and Methods

The study was conducted in Katari community of Kachia Local Government Area (LGA) in Kaduna State, northwest Nigeria. The community was randomly selected from the 22 communities that make up the LGA. The estimated population of the community is 6,572 [18]. Most of the inhabitants are small scale farmers and traders. Health care in the community is provided mainly by traditional healers, patent medicine shops, and a primary healthcare centre in the community. The nearest General Hospital to the community is 30 Km away and serves as a referral centre to Katari's PHC.

Adolescents in the community were defined as regular inhabitants of the community whose age ranged from 10 to 19 years [19]. Age was determined by evidence of birth records or corroborated oral evidence. AWE were identified via a house-to-house epilepsy survey in the community. For epidemiological surveys, and in this study, epilepsy was defined as recurrent unprovoked epileptic seizures occurring at least 24 hours apart [20, 21].

After a house-to-house survey is conducted by the authors a total of 18 adolescents were identified as having epilepsy out of a total adolescent population of 1,708 [22]. Parameters of AWE assessed included age, sex, social class, educational status, clinical features of epilepsy, current and past treatment options utilized, history of central nervous system infections, perinatal history, and assessment of intelligence using Raven's Progressive Matrix. Social class distribution was determined using the Ogunlesi et al. classification [23]. The Family APGAR tool was used in assessing perceived family function by the AWE. The tool has been validated and found to be reliable in assessing family function [24]. The Family APGAR Score uses the family's member perception of satisfaction to assess 5 dimensions of family functioning, namely, Adaptability, Partnership, Growth, Affection, and Resolve [24]. Each parameter is assessed on a 3-point scale ranging from 0 (hardly ever) to 2 (almost always). The final grading is based on the cumulative score and is graded as 0–3 (highly dysfunctional family), 4–6 (moderately dysfunctional family), and 7–10 (highly functional family) [24].

Ethical approval for the study was obtained from the Research Ethics Committee of the FNPH Kaduna. Informed consent was obtained from the community and household heads in Katari community and the AWE who were 18 years old or older while assent was obtained from those younger. A month's dose of the AED phenobarbitone was provided to all the AWE who needed to be on AED therapy but were not and they were referred to the Child and Adolescent Mental Health (CAMH) Unit of the Federal Neuropsychiatric Hospital (FNPH), Kaduna, for further management. The CAMH Unit has adequate facilities for the management of epilepsy. Furthermore, the AWE and their families were introduced to a nongovernmental organization that supports the management of persons with epilepsy and their families.

Data was analyzed using Epi Info 3.5.3 statistical package. Chi-square test, with Yates' correction where applicable, was used in determining the relationship between the characteristics of the AWE and their perception of family function. A *P* value less than 0.05 was regarded as significant.

## 3. Results

Adolescents (*n* = 1708) constituted 26% of the community's population out of which 18 (10.5/1000) had epilepsy. The age range of the subjects was 11–19 years (mean 16.7 ± 2.6 years) with a male preponderance (15, 83.3%) (Table 1). All 3 females were married but 2 were currently separated from their husbands as a result of inability of their spouses to cope with their epilepsy. All (18, 100%) were in the lower (classes IV and V) social classes. Also all had had a primary education, 4 (22%) had completed a secondary education, and 4 (22%) had experienced school rejection as a result of having epilepsy.

All the AWE had active epilepsy with generalized seizures occurring at least once weekly and monthly in 10 and 8 AWE, respectively. The seizures were tonic-clonic (12, 66.7%), tonic (5, 27.8%), and absence (1, 0.5%), respectively. All were currently on traditional herbal medication. None was currently on orthodox medical treatment even though 3 (16.7%) had accessed orthodox medical treatment, at the community's Primary Healthcare Centre (PHC), in the past. Those who visited the PHC were further referred to the nearest General Hospital for further management. After initial visits to the General Hospital, where they were seen and commenced on oral phenobarbitone, all three stopped subsequent follow-up visits and AED treatment due to financial constraints. The family was the only source of care and support for the AWE.

There was a family history of epilepsy in 8 (44.4%) of the AWE. Two (11.1%) of them were siblings, and in the remaining 6 (88.9%) AWE there was history of epilepsy in adult relatives.

There was no history of trauma to the head or that suggestive of a central nervous system infection in the AWE.

Most (14, 77.8%) of the AWE were delivered at home by traditional birth attendants while the others (4, 22.2%) were delivered in the community's PHC. The deliveries and the neonatal period of all the AWE were described as normal.

Assessment of intelligence using Raven's Progressive Matrix indicated normal intelligence in all the AWE.

Moderate family dysfunction (Family APGAR Score <7 and >3) was indicated by 15 (83.3%) of the AWE while the others (3, 16.7%) acknowledged living in a highly functional (Family APGAR > 7) setting. None indicated being in a highly dysfunctional setting. The AWE that are siblings, and all the female AWE, indicated moderate family dysfunction. The strongest perception of family function was in Adaptability while the weakest was with Growth (Table 2). The indication of family dysfunction was significant (*P* < 0.05) in the older (age 14–19 years) AWE when compared with the younger AWE (11–13 years) in the study (Table 3).

## 4. Discussion

The perception of family function by the AWE was predominantly dysfunctional particularly with regard to satisfaction in the Growth, Affection, and Partnership parameters of the Family APGAR. The satisfaction with the Adaptability parameter, one that assesses the family as an institution that could easily adapt and be referred to at the time of a need, was the strongest attribute of family function indicated. The study also identified the family as the only source of care and support for the AWE.

Studies have indicated that families of children with epilepsy have generally fared worse than controls [12]. Thornton et al. [12] reported poorer scores with regard to role performance among families of children with epilepsy. Additionally, these families have been found out to have lower levels of communication, family social support, and financial wellbeing [25]. Coping with frequently occurring epileptic seizures, widened epilepsy treatment gap, and lack of institutional support, which were all observed in this study, could be quite challenging to families and impair their functioning [26]. Conversely in a study by Adewuya et al. [7] AWE rated their families as highly functional. However the study was conducted in an urban setting where most of the AWE were in more affluent mid and upper social classes and had access to AEDs as well as quality epilepsy care [7].

Studies from Nigeria indicate that AWE have perceptions of shame, depression, lowered self-esteem, and stigmatization [7–9]. Other reports also indicate poor knowledge of epilepsy, impaired HRQoL, and negative attitudes among AWE [10, 17]. To the best of our knowledge this is the first study, from northern Nigeria, that focuses specifically on the perception of AWE on family function. However the perceptions of AWE on social relationships could be gleaned from studies concerning their HRQoL. In one of such studies from southwest Nigeria, findings reported by Lagunju et al. [8] included family disruption in 50% of the families with a child who had epilepsy and marital disharmony leading to divorce in 7.6% of them. Other findings in that study included the expression of family neglect by siblings of affected children and caregivers' loss of income and financial benefits. Also the feelings of stigma, being fed up with life, inadequacy and lowered self-esteem were expressed by the children with epilepsy [8]. Also reports by Eseigbe et al. [26] and Nuhu et al. [27], from northern Nigeria, indicate stigma and socioeconomic challenges as well as significant caregiver burden in families providing care for epilepsy in childhood. The family settings in these studies are similar, and some even fairer, when compared to those in our study. The epilepsy related family burden and challenges highlighted in these studies could have influenced the poor perception of family functioning, particularly in the Family APGAR parameters of partnership (satisfaction with the way my family shares my problem with me) and Growth (satisfaction with my family's acceptance and support in my taking upon new activities), by the AWE in our study. Poor knowledge and misconceptions about epilepsy which are also common in the African region often result in fear, stigma, and discrimination among caregivers and other family members towards persons with epilepsy [2, 8, 28]. These resultant negative attitudes by family members could have contributed to the dissatisfaction associated with the Growth and Affection (satisfaction with family's expression of affection and regards towards my feelings) parameters, which were indicated by most of the AWE. The more positive responses indicated with regard to Adaptability (satisfaction with turning to my family whenever troubled) and Resolve (satisfaction with the way family and I share time together) underscores the dominant role of families in the provision of care for the AWE [26]. This role, as well as limited health and social support services for persons with epilepsy, has been reported severally from low and middle income countries (LMICs) where the burden of epilepsy is high [2].

Age was the only characteristic of the AWE that was significantly associated with perceived family dysfunction in this study. The likelihood of being more critical of relationships and having lofty expectations which increases with age could explain the significant dysfunctional appraisal by the older adolescents in the study. This could further explain the poor scores associated with the Adaptability, Growth, and Affection parameters of the Family APGAR. The other sociodemographic variables of the AWE did not significantly influence the perception of family function. The small sample size of the AWE and the relative homogeneity of their existing circumstances could have been accountable. Also the characteristics of epilepsy did not significantly influence the perceptions of the AWE. Epilepsy variables, particularly its severity, have been observed to correlate with greater perceived impact on the family unit [29]. The possibility of severe forms of epilepsy fostering more family attention in those affected could have had a positive influence on the perceptions of the AWE. This could have countered the negative perceptions of family function that could have arisen from the severity of the disease. Thornton et al. [12] also reported the nonsignificance of epilepsy variables on family function even though they acknowledged that the number of those with intractable epilepsy in their study was very small.

Generally the perspectives of adolescents on issues are vital to their coping skills and health or help seeking behavior [15]. These perspectives could also influence development of abnormal behavior, psychopathology, substance abuse, and an increased risk of mortality [15]. Family function has been demonstrated to be very important in predicting adaptive, cognitive, and behavioral function in childhood epilepsy [12]. Poor academic achievement, impaired HRQoL, behavioral abnormalities, and an increased burden of disease are adverse epilepsy outcomes that have been associated with family dysfunction [8, 12, 30]. Consequently the perception and reality of family dysfunction among AWE could have grave implications for epilepsy outcomes particularly in regions where these outcomes are already grim. Dissemination of appropriate epilepsy information to the community and persons with epilepsy, improved access to treatment, provision of social support for affected families, and protection of the rights of persons with epilepsy could all strengthen family functioning and help improve its perception by AWE with desirable outcomes.

## 5. Limitations

The perceptions in this study were those of AWE in a rural community and of a predominantly low socioeconomic status; the perceptions of AWE in urban settings and in upper socioeconomic settings which have increased access to epilepsy care and information could be different. Also the role of family variables such as family size and presence of other chronic illnesses was not included in our study. However, majority of persons with epilepsy in LMICs live in similar settings as the AWE in this study.

## 6. Conclusion

The perception of living in a dysfunctional family setting was indicated by most of the AWE in a rural Nigerian community. Weak perception of satisfaction with family function was mainly with Growth (satisfaction with family's acceptance and support towards my taking on new activities) and Affection (satisfaction with family's expression of affection and regards towards my feelings) parameters of the Family APGAR assessment tool while the strongest was with Adaptability (satisfaction with turning to my family whenever troubled). The study highlights the need to address the role of the family in the provision of comprehensive epilepsy care particularly in regions with poor epilepsy outcomes.

## Figures and Tables

**Table 1:** Age and sex distribution of the AWE.

Age group (years)	Sex		Number of AWE (%)
	M (%)	F (%)	
11–13	4 (26.7)	0	4 (22.2)
14–16	2 (13.3)	0	2 (11.1)
17–19	9 (60)	3 (100)	12 (66.7)

Total	15 (100)	3 (100)	18 (100)

AWE: adolescents with epilepsy.

**Table 2:** Distribution of optimal score (2, almost always) in the Family APGAR [11] Scores of the AWE.

Family APGAR parameter [24]	Family APGAR interpretation [24]	Number of AWE indicating optimal score (%)
Adaptability	I can turn to my family for help when something is troubling me	13 (72.2)
Partnership	I am satisfied with the way my family shares my problem with me	4 (22.2)
Growth	I am satisfied that my family accepts and supports my wishes to take on new activities	2 (11.1)
Affection	I am satisfied with the way my family expresses affection and regard to my emotions, anger, sorrow, and love	3 (16.7)
Resolve	I am satisfied with the way my family and I share time together	10 (55.6)

[24].

**Table 3:** Relationship between some variables of the AWE and their perception of family function (Family APGAR).

AWE variables	Number of AWE *n* = 18 (%)	Family APGAR		*P* value
		Functional	Dysfunctional	
		*n* = 3 (%)	*n* = 15 (%)	
Age (years)				
>14	14 (77.8)	0	14 (93.3)	0.01^*^
<14	4 (22.2)	3 (100)	1 (6.7)	
Sex				
Male	15 (83.3)	3 (100)	12 (80)	0.40^*^
Female	3 (16.7)	0	3 (20)	
Seizure frequency				
Weekly	10 (55.6)	3 (100)	7 (46.7)	0.29^*^
Monthly	8 (44.4)	0	8 (53.3)	
School rejection				
Yes	4 (22.2)	2 (66.7)	2 (13.3)	0.21^*^
No	14 (77.8)	1 (33.3)	13 (86.7)	
2nd school completion				
Yes	4 (22.2)	0	4 (26.7)	0.80^*^
No	14 (77.8)	3 (100)	11 (73.3)	
Family history (epilepsy)				
Positive	8 (44.4)	2 (66.7)	6 (40)	0.83^*^
Negative	10 (55.6)	1 (33.3)	9 (60)	

AWE: adolescents with epilepsy; ^*^: with Yates' correction.
